# Human single-neuron correlates of somatosensation

**DOI:** 10.64898/2026.10.07.757446

**Published:** 2026-11-02

**Authors:** Bingchuan Liu, David Bjanes, Liang She, Kelsie Pejsa, Brian Lee, Charles Y. Liu, Richard A. Andersen

## Abstract

Understanding the neural correlates of consciousness poses a central challenge in neuroscience, with longstanding debate over which cortical areas underlie subjective sensory perception. In the tactile domain, posterior parietal cortex (PPC) and primary somatosensory cortex (S1) are candidate cortical substrates for neural correlates of consciousness due to their crucial roles in somatosensory processing. Yet whether and how these regions contribute to conscious tactile perception remains unclear. Here, to address this issue, we leveraged chronic intracortical recordings to simultaneously monitor large ensembles of single neurons in grasp related cortical areas from three participants with tetraplegia while natural vibrotactile stimuli were delivered to a sensate region of the upper limb. We demonstrate that an orchestration of neural activity emerged across the grasp-related parietal-frontal network at near-threshold perception. Within this distributed network, we then focused on PPC and S1. While S1 responses closely tracked the amplitude of the physical stimulus, PPC activity covaried more strongly with awareness and confidence. This single-neuron evidence reveals PPC and S1 have complementary neural codes with PPC transforming physical stimulation into conscious perception whereas S1 is more sensitive to stimulus amplitude.

## Full Text Availability

The license terms selected by the author(s) for this preprint version do not permit archiving in PMC. The full text is available from the preprint server.

